# Beliefs About the Health Effects of Smoking Among Adults in the United States

**DOI:** 10.1177/10901981211004136

**Published:** 2021-04-17

**Authors:** Sarah D. Mills, Christopher A. Wiesen

**Affiliations:** 1 Department of Health Behavior, Gillings School of Global Public Health, University of North Carolina, Chapel Hill, NC, USA; 2 Lineberger Comprehensive Cancer Center, University of North Carolina, Chapel Hill, NC, USA; 3 Odum Institute, University of North Carolina, Chapel Hill, NC, USA

**Keywords:** health belief model, health disparities, health equity, smoking and tobacco use, substance use

## Abstract

The majority of U.S. adults believe that smoking is a cause of lung cancer, but research suggests that the percentage of adults who believe smoking causes other types of cancers and chronic disease is lower. This study examines the correlates of beliefs about several established health effects of smoking in a nationally representative sample of U.S. adults. Data for this study come from Wave 4 of the Population Assessment of Tobacco and Health Study conducted from December 2016 to January 2018. Participants responded to questions assessing their beliefs about the health effects of smoking. Logistic regression models were used to examine the relationship between beliefs about the health effects of smoking and sociodemographic characteristics (smoker status, age, sex, education, race/ethnicity), exposure to antitobacco campaigns, smokers’ health, and nicotine dependence. The percentage of U.S. adults who endorsed a health effect can be caused from smoking ranged from 56.4% for blindness to 97.4% for lung disease. Respondents who were older, less educated, current or former smokers, and had less exposure to antitobacco campaigns were generally less likely (*p* < .05) to endorse that an established health effect was caused by smoking. Smokers with lower nicotine dependence and worse health were generally more likely (*p* < .05) to endorse that an established health effect was caused by smoking. In summary, knowledge about the health effects of smoking varies across health conditions. Public health would benefit from campaigns targeting segments of the population with less knowledge about the health effects of smoking.

Significant progress has been made in the study of the health effects of smoking. The International Union Against Cancer first reported that there was a dose-dependent relationship between smoking and lung cancer in 1952 (Proctor, 2011). The 1964 Surgeon General’s Report concluded that smoking was a cause of lung cancer and laryngeal cancer in men, a probable cause of lung cancer in women, and the most frequent cause of chronic bronchitis (U.S. Public Health Service, 1964). Studies now indicate that smoking can harm nearly every organ in the body (U.S. Department of Health and Human Services, 2014).

According to the 2014 Surgeon General’s Report, smoking is causally linked with several cancers, including cancers of the lung, bladder, stomach, liver, kidney, pancreas, larynx, oropharynx, and the esophagus (U.S. Department of Health and Human Services, 2014). Smoking also increases the likelihood of chronic diseases, including stroke, blindness, heart disease, and diabetes (U.S. Department of Health and Human Services, 2014). Among women, smoking has negative reproductive effects, reducing fertility and increasing risk of ectopic pregnancy. Exposure to secondhand smoke is causally linked to nasal irritation, stroke, lung cancer, and heart disease among adults, adverse reproductive effects among women, and health conditions among children such as middle ear disease, lower respiratory illness, impaired lung function, and sudden infant death syndrome (U.S. Department of Health and Human Services, 2014).

In parallel with scientific progress, the percentage of adults who report that smoking is harmful to health has increased since the middle of the 20th century (Krosnick et al., 2006). In the 1950s, 42% of individuals living in the United States believed that smoking was a cause of lung cancer (Marshall, 2014). The vast majority (>90%) believed that smoking was a cause of lung cancer by the early 1990s (Marshall, 2014). Studies suggest that a smaller percentage of adults believe that smoking causes other types of cancer or chronic disease; however, this research is dated and most studies are limited to samples of smokers. A nationally representative study of U.S. adult smokers conducted in 2002 examined beliefs about different health effects of smoking (Hammond et al., 2006). The majority (94%) of smokers reported that smoking causes lung cancer in smokers. Sixty-eight percent and 73% of smokers reported that smoking causes lung cancer in nonsmokers and stroke in smokers, respectively. Only 34% of smokers reported that smoking causes impotence (Hammond et al., 2006). In a 2005 study of smokers in Greater New Haven, Connecticut, 99% of smokers reported that lung cancer was caused or made worse by smoking (Oncken et al., 2005). On the other hand, 68%, 48%, and 44% reported that head and neck cancers, cervical cancer, and reproductive difficulties, respectively, are caused or made worse by smoking. This is of concern because risk-minimizing beliefs are associated with smoking onset among youth and lower quit intentions among smokers (Borland et al., 2009; Krosnick et al., 2006). According to the health belief model, susceptibility to harm is a critical motivator of behavior change (Rosenstock, 1974). A smoker who perceives their susceptibility to several diseases as high may be more likely to quit than one who perceives their susceptibility as low.

In addition, studies indicate that age, sex, education, race/ethnicity, and smoker status are associated with beliefs about the health effects of smoking (Ayanian & Cleary, 1999; Brownson et al., 1992; Klesges et al., 1988; Oncken et al., 2005; Rutten et al., 2008). Those who are older, less educated, and current smokers less frequently endorse established health risks of smoking (Ayanian & Cleary, 1999; Brownson et al., 1992; Klesges et al., 1988; Oncken et al., 2005; Rutten et al., 2008). These associations may contribute to demographic disparities in tobacco use. Relationships between sex and race/ethnicity and health beliefs have been inconsistent, with studies finding nonsignificant and significant relationships (Brownson et al., 1992; Klesges et al., 1988; Rutten et al., 2008). The most recent study using nationally representative data from the 2003 Health Information National Trends Survey found no differences between men and women or by race/ethnicity in beliefs about how much smoking increases the risk of cancer (Rutten et al., 2008).

In addition to sociodemographic characteristics, other factors may play an important role in beliefs about the health effects of smoking. Studies suggest that public health campaigns that focus on the health consequences of smoking are associated with perceptions about health risks, but research is not consistent (Allen et al., 2015; Durkin et al., 2012; Huang et al., 2015; Kranzler et al., 2017). Huang et al. (2015) examined the impact of the 2013 *Tips From Former Smokers* (*Tips*) campaign, a national mass media campaign funded by the Centers for Disease Control and Prevention (CDC) to encourage quitting. The *Tips* campaign includes smokers’ testimonials describing how their lives have been affected by smoking-related diseases and conditions (Huang et al., 2015). Recall of campaign advertisements was positively associated with knowledge about campaign-targeted health effects for some conditions discussed in the campaign, but not others (Huang et al., 2015). Similarly, Richardson et al. (2010) found that awareness of the *Truth* campaign, a national tobacco countermarketing campaign, was associated with agreement with some, but not all, of the message content of the campaign. The *Tips* campaign, which airs annually, as well as other antitobacco campaigns, may play a key role in beliefs about the health effects of smoking.

Among smokers, those who have had greater damage to their health as a result of their tobacco use may be more knowledgeable about smoking’s health effects. On the other hand, cognitive dissonance theory suggests that, given the difficulty of quitting, smokers may downplay the health consequences of smoking, even if their health has been damaged, in an effort to reduce cognitive dissonance (Festinger, 1957; Fotuhi et al., 2013). Similarly, fewer smokers with higher levels of nicotine dependence, a proxy for addiction, may correctly endorse established health effects of smoking. Smokers with high levels of addiction may be more likely to adjust their beliefs to reduce potential dissonance (Fotuhi et al., 2013).

This study examines beliefs about established health effects of smoking in a nationally representative sample of U.S. adults using data from the Population Assessment of Tobacco and Health (PATH) Study. In addition, this study examines relationships between health beliefs and sociodemographic characteristics as well as the relationship between health beliefs and exposure to antitobacco campaigns, smokers’ health, and nicotine dependence. No recent national study has examined beliefs about smoking’s health effects for several established types of harm. Examining beliefs about the health effects of smoking may indicate if more education is needed, for whom, and areas of focus for smoking prevention efforts.

## Method

The PATH Study is a longitudinal, nationally representative cohort study of tobacco use behavior and beliefs and tobacco-related health outcomes among civilian, noninstitutionalized youth and adults in the United States (Hyland et al., 2017). The PATH Study is conducted through a collaboration between the National Institute on Drug Abuse and the Food and Drug Administration’s Center for Tobacco Products. PATH uses a four-stage, stratified probability sample design (U.S. Department of Health and Human Services, 2020). Adults are oversampled for young adults (18–24 years), African Americans, and tobacco users. Data for the present study come from adult (18+ years) interviews in Wave 4 of the Study. The Wave 4 survey was administered from December 2016 to January 2018. Response rates for adult participants were 73.5%. PATH collects baseline and follow-up data through in-person audio computer-assisted interviews (ACIS). ACIS is a method for data collection in which a respondent listens to prerecorded questions and responds by selecting answers on a touch screen or keypad. Biospecimens are also collected but were not used in the present study. For a detailed description of the PATH Study, see Hyland et al. (2017).

### Measures

#### Sociodemographic Characteristics

The following variables and categories were employed: age (18–24, 25–34, 35–64, 65+ years), sex (male, female), education (high school graduate or less, some college or associates degree, and bachelor’s or advanced degree), race/ethnicity (non-Hispanic White, non-Hispanic Black, non-Hispanic Other, and Hispanic), and smoker status (current smoker, former smoker, never smoker). Current smokers were defined as respondents who smoked at least 100 cigarettes in their lifetime and currently smoke every day or some days. Former smokers were defined as respondents who smoked at least 100 cigarettes in their lifetime and currently do not smoke at all. All other respondents were classified as never smokers (i.e., respondents who had not smoked at least 100 cigarettes in their lifetime).

#### Beliefs About the Health Effects of Smoking

Respondents were asked to answer the following question: “Based on what you believe, how much do you agree or disagree with the following statements? Smoking can cause [health effect] in smokers.” The following health effects were examined: diabetes, liver cancer, stroke, lung cancer, heart disease, blindness, poor circulation, mouth cancer, bladder cancer, and lung disease. Three questions were also asked about the health effects of secondhand smoke (i.e., “Smoking can cause lung disease in nonsmokers from secondhand smoke,” “Smoking can cause heart attack in nonsmokers from secondhand smoke,” and “Smoking can cause harm to fetuses during pregnancy from secondhand smoke”). Response options were on a five-item scale ranging from “Strongly disagree” to “Strongly agree.” For the present study, respondents who reported that they “Strongly agree” or “Agree” were considered to believe that the health effect was caused by smoking.

#### Antitobacco Campaigns

Respondents indicated the frequency with which they have viewed an antitobacco campaign by responding to the question, “In the past 12 months, how often did you see an antitobacco advertising campaign?” Response options were “Never,” “Rarely,” “Sometimes,” and “Often.”

#### Dependence

Nicotine dependence was assessed among current smokers by the self-reported length of time to first cigarette after waking. Nicotine dependence was categorized into two categories: less than 30 minutes and greater than or equal to 30 minutes, which reflect higher and lower levels of dependence, respectively.

#### Health

Current and former smokers indicated the extent to which tobacco products have damaged their health by responding to the question, “To what extent, if it all, has using/your past use of tobacco damaged your health?” Response options were “Not at all,” “A little,” “Somewhat,” and “A lot.” This item was examined only among current smokers in the present study.

### Data Analysis

The sample was limited to adults who responded to all questions about the health effects of smoking and had no missing data on smoker status, education, age, or the item about campaign viewing. This resulted in removing 4.6% (*n* = 1,533) of participants, leaving a total sample size of 32,111. The present study used the imputed variables for sex, race, and ethnicity provided by the PATH Study. Imputation methods are described elsewhere (U.S. Department of Health and Human Services). A subsample of current smokers was also used for analysis. For this subsample, current smokers were limited to those who also had no missing data on nicotine dependence and an item querying about damaged health due to tobacco use. This resulted in removing 0.5% (*n* = 43) of all current smokers, leaving a total sample size of 9,406 participants.

First, descriptive statistics of the sample were examined separately for current smokers, former smokers, and never smokers. Next, the percentage of respondents who endorsed each of the 13 health effects of smoking was obtained in the total sample and according to smoker status. In the total sample, logistic regression models were used to assess for significant differences in health beliefs according to smoker status. In addition, in adjusted models, logistic regression was used to examine how beliefs about the health effects of smoking were associated with sociodemographic characteristics (smoker status, age, sex, education, and race/ethnicity) and frequency of viewing antitobacco campaigns. Among a subsample of current smokers, logistic regression was used to examine how beliefs about the health effects of smoking are associated with nicotine dependence and the extent to which tobacco products have damaged one’s health, controlling for sociodemographic characteristics.

Analyses were conducted in SAS Version 9.4 using repeated replication methods to account for the complex survey design. To obtain nationally representative estimates, the “Wave 4 Cohort Cross-Sectional Weights” were used. The full sample and replicate weights were used for the descriptive statistics and logistic regression models. The balance repeated replication method with Fay’s adjustment set to 0.3 was used to compute variances.

## Results

Descriptive statistics are in Table 1. In the present study, 17.8% of the sample were current smokers.

**Table 1.** Sample Characteristics, Population Assessment of Tobacco and Health Study (Wave 4).

Characteristic	Current smokers		Former smokers		Never smokers	
	Unweighted frequency (*N* = 9,449)	Weighted percentage	Unweighted frequency (*N* = 5,863)	Weighted percentage	Unweighted frequency (*N* = 16,799)	Weighted percentage
Age (years)						
18–24	1,655	10.1	639	3.2	8,534	16.6
25–34	2,371	24.6	1,191	11.6	3,068	18.5
35–64	4,766	56.9	2,924	52.6	4,266	47.9
65+	657	8.3	1,109	32.6	1,111	16.9
Sex						
Male	4,692	53.8	3,169	53.0	7,863	44.4
Female	4,757	46.2	2,694	47.0	8,936	55.6
Education						
High school graduate or less	5,214	56.8	1,940	37.4	6,480	34.4
Some college or associates degree	3,272	32.5	2,233	32.7	6,007	30.1
Bachelor’s or advanced degree	963	10.7	1,690	29.9	4,312	35.5
Race/ethnicity						
Non-Hispanic White	6.025	68.4	4,081	76.1	8,288	59.1
Non-Hispanic Black	1,389	13.3	548	6.9	2,911	12.9
Non-Hispanic Other	734	5.9	396	6.0	1,430	9.8
Hispanic	1,301	12.4	838	11.0	4,170	18.2

### Beliefs About the Health Effects of Smoking

Beliefs about the effects of smoking varied across the health effects examined (Table 2). The percentage of U.S. adults who correctly endorsed a health effect can be caused from smoking ranged from 52.8% for blindness to 97.4% for lung disease. For all 13 health effects examined, compared with never smokers, current smokers were less likely (*p* < .05) to report that a health effect was caused by smoking. For most health effects, former smokers were less likely to report that a health effect was caused by smoking as compared with never smokers.

**Table 2.** Percentage of U.S. Adults Who Believe in Established Health Effects of Smoking.

Characteristic	Diabetes	Liver cancer	Stroke	Lung cancer	Heart disease	Blindness	Poor circulation	Mouth cancer	Bladder cancer	Lung disease	Lung disease in nonsmokers	Heart attack in nonsmokers	Harm to fetus
Total sample (*N* = 32,111)	56.4	69.9	90.5	96.9	94.7	52.8	88.1	93.2	64.1	97.4	88.4	73.6	90.1
Smoker status													
Current smoker (*n* = 9,449)	47.0*	56.7*	83.9*	91.1*	88.6*	44.4*	79.7*	83.9*	52.8*	92.6*	73.9*	59.3*	78.4*
Former smoker (*n* = 5,863)	54.4*	66.1*	92.1	97.9	96.1	50.2*	89.4	93.9*	61.7*	98.3	88.8*	74.3*	89.1*
Never smoker (*n* = 16,799)	60.1	75.4	91.9	98.2	96.0	56.4	90.2	95.8	68.5	98.4	92.6	77.6	93.9

*Note*. Values presented are weighted percentages. Sample size presented is unweighted. For comparisons across smoker status groups, “Never smoker” is the reference group.

* *p* < .05.

### Sociodemographic Differences in Health Beliefs

#### Smoker Status

In adjusted models, smoker status was significantly (*p* < .05) associated with beliefs about each health effect examined. Across health effects, current smokers were 0.2 (95% confidence interval [CI; 0.2, 0.3]) to 0.6 (95% CI [0.5, 0.6]) times as likely to endorse that smoking can cause a given health effect as compared with nonsmokers (Table 3). In addition, for most health effects, former smokers were 0.7 [0.6, 0.7] to 0.9 [0.8, 1.0] times as likely to endorse that smoking can cause a given health effect as compared with never smokers.

**Table 3.** Correlates of Beliefs About the Health Effects of Smoking.

Characteristic	Diabetes	Liver cancer	Stroke	Lung cancer	Heart disease	Blindness	Poor circulation	Mouth cancer	Bladder cancer	Lung disease	Lung disease in nonsmokers	Heart attack in nonsmokers	Harm to fetus
Smoker status													
Current smoker	**0.6 [0.5, 0.6]**	**0.4 [0.4, 0.5]**	**0.5 [0.4, 0.5]**	**0.2 [0.2, 0.3]**	**0.3 [0.3, 0.4]**	**0.6 [0.6, 0.7]**	**0.5 [0.4, 0.5]**	**0.3 [0.2, 0.3]**	**0.5 [0.5, 0.6]**	**0.2 [0.2, 0.3]**	**0.3 [0.2, 0.3]**	**0.4 [0.4, 0.5]**	**0.3 [0.2, 0.3]**
Former smoker	**0.8 [0.8, 0.9]**	**0.7 [0.6, 0.7]**	1.0 [0.9, 1.2]	0.9 [0.7, 1.3]	1.0 [0.8, 1.3]	**0.8 [0.8, 0.9]**	0.9 [0.8, 1.1]	**0.8 [0.6, 0.9]**	**0.7 [0.7, 0.8]**	0.9 [0.7, 1.3]	**0.7 [0.6, 0.8]**	**0.9 [0.8, 1.0]**	**0.7 [0.6, 0.8]**
Never smoker	Ref	Ref	Ref	Ref	Ref	Ref	Ref	Ref	Ref	Ref	Ref	Ref	Ref
Age (years)													
18–24	Ref	Ref	Ref	Ref	Ref	Ref	Ref	Ref	Ref	Ref	Ref	Ref	Ref
25–34	**1.1 [1.0, 1.2]**	**1.1 [1.0, 1.2]**	**1.2 [1.1, 1.5]**	0.9 [0.7, 1.2]	1.0 [0.9, 1.2]	**1.2 [1.1, 1.3]**	0.9 [0.8, 1.0]	1.1 [1.0, 1.3]	1.0 [0.9, 1.2]	1.1 [0.9, 1.4]	**1.3 [1.2, 1.5]**	**1.3 [1.2, 1.5]**	0.9 [0.8, 1.1]
35–64	**1.1 [1.0, 1.2]**	**1.1 [1.0, 1.1]**	**1.3 [1.1, 1.5]**	**0.6 [0.6, 0.9]**	**1.2 [1.1, 1.3]**	**1.1 [1.0, 1.2]**	**0.9 [0.8, 1.0]**	**0.8 [0.7, 0.9]**	**1.2 [1.0, 1.4]**	1.1 [0.8, 1.4]	1.0 [0.9, 1.2]	**1.2 [1.1, 1.3]**	**0.7 [0.6, 0.8]**
65+	**0.9 [0.8, 1.0]**	**0.9 [0.8, 0.9]**	**0.8 [0.7, 0.9]**	**0.5 [0.4, 0.7]**	0.9 [0.7, 1.0]	0.9 [0.8, 1.0]	**0.7 [0.6, 0.9]**	**0.8 [0.7, 0.9]**	**1.2 [1.0, 1.4]**	1.1 [0.8, 1.5]	1.0 [0.8, 1.2]	**1.3 [1.1, 1.4]**	**0.5 [0.5, 0.7]**
Sex													
Male	Ref	Ref	Ref	Ref	Ref	Ref	Ref	Ref	Ref	Ref	Ref	Ref	Ref
Female	1.0 [0.9, 1.0]	**1.2 [1.1, 1.3]**	**1.2 [1.0, 1.3]**	0.9 [0.8, 1.1]	1.0 [0.8, 1.1]	1.0 [0.9, 1.1]	1.1 [1.0, 1.2]	**1.4 [1.2, 1.5]**	**1.0 [0.9, 1.1]**	1.1 [0.9, 1.2]	**1.4 [1.3, 1.6]**	**1.1 [1.1, 1.2]**	**1.2 [1.1, 1.4]**
Education													
High school graduate or less	Ref	Ref	Ref	Ref	Ref	Ref	Ref	Ref	Ref	Ref	Ref	Ref	Ref
Some college or associates degree	0.9 [0.9, 1.0]	0.9 [0.8, 1.0]	**1.3 [1.2, 1.5]**	**1.7 [1.5, 2.1]**	**1.6 [1.3, 1.8]**	1.0 [0.9, 1.0]	**1.6 [1.3, 1.8]**	**1.7 [1.5, 2.0]**	1.0 [0.9, 1.0]	**1.8 [1.5, 2.2]**	**1.3 [1.1, 1.4]**	1.1 [1.0, 1.2]	**1.3 [1.2, 1.5]**
Bachelor’s or advanced degree	1.1 [1.0, 1.2]	0.9 [0.8, 1.2]	**2.2 [1.8, 2.6]**	**4.2 [2.9, 6.0]**	**2.2 [1.7, 2.7]**	1.0 [1.0, 1.1]	**2.4 [2.0, 2.9]**	**3.1 [2.5, 3.8]**	1.0 [1.0, 1.2]	**4.9 [3.4, 7.2]**	**2.1 [1.8, 2.5]**	**1.1 [1.1, 1.4]**	**2.0 [1.7, 2.4]**
Race/ethnicity													
White	Ref	Ref	Ref	Ref	Ref	Ref	Ref	Ref	Ref	Ref	Ref	Ref	Ref
Black	**1.1 [1.0, 1.3]**	**1.4 [1.3, 1.6]**	**0.7 [0.6, 0.8]**	**0.7 [0.5, 0.8]**	**0.7 [0.6, 0.9]**	**1.4 [1.3, 1.6]**	**0.6 [0.6, 0.8]**	**0.8 [0.7, 0.9]**	**1.3 [1.2, 1.4]**	**0.6 [0.5, 0.8]**	1.0 [0.9, 1.1]	**1.2 [1.1, 1.3]**	**1.5 [1.3, 1.8]**
Other	1.0 [0.8, 1.1]	1.1 [0.9, 1.3]	0.8 [0.6, 1.0]	**0.6 [0.4, 1.0]**	**0.6 [0.4, 0.8]**	1.2 [1.0, 1.4]	0.7 [0.6, 0.9]	**0.8 [0.6, 1.0]**	1.0 [0.9, 1.2]	**0.6 [0.4, 0.9]**	1.0 [0.8, 1.2]	1.0 [0.8, 1.2]	1.2 [1.0, 1.6]
Hispanic	**1.2 [1.1, 1.3]**	**1.3 [1.2, 1.4]**	**0.6 [0.5, 0.7]**	0.9 [0.7, 1.1]	**0.6 [0.6, 0.8]**	**1.4 [1.3, 1.6]**	**0.8 [0.7, 0.9]**	**1.2 [1.1, 1.5]**	**1.1 [1.0, 1.2]**	**0.8 [0.6, 0.9]**	**1.5 [1.3, 1.7]**	**1.2 [1.1, 1.4]**	**1.7 [1.4, 2.0]**
Antitobacco campaign													
Never	Ref	Ref	Ref	Ref	Ref	Ref	Ref	Ref	Ref	Ref	Ref	Ref	Ref
Rarely	0.9 [0.9, 1.0]	1.0 [0.9, 1.1]	**1.2 [1.0, 1.5]**	**1.1 [0.9, 1.2]**	1.2 [1.0, 1.5]	**0.9 [0.8, 1.0]**	**1.3 [1.1, 1.5]**	1.2 [1.1, 1.4]	0.9 [0.8, 1.0]	**1.7 [1.3, 2.1]**	1.0 [0.9, 1.1]	0.9 [0.9, 1.1]	**1.2 [1.0, 1.4]**
Sometimes	1.1 [1.0, 1.2]	1.0 [0.9, 1.1]	**1.4 [1.2, 1.7]**	**1.1 [0.9, 1.2]**	**1.5 [1.3, 1.8]**	1.0 [1.0, 1.2]	**1.6 [1.4, 1.8]**	**1.6 [1.4, 1.9]**	1.0 [0.9, 1.1]	**2.0 [1.5, 2.5]**	1.0 [0.9, 1.2]	1.1 [0.9, 1.2]	1.2 [1.0, 1.3]
Often	1.1 [1.0, 1.3]	1.1 [0.9, 1.2]	**1.7 [1.3, 2.1]**	**1.2 [1.0, 1.5]**	**1.5 [1.2, 1.8]**	1.1 [1.0, 1.2]	**1.9 [1.6, 2.2]**	**1.8 [1.5, 2.2]**	**1.1 [1.0, 1.3]**	**1.9 [1.4, 2.7]**	1.0 [0.8, 1.2]	**1.1 [1.0, 1.3]**	1.1 [1.0, 1.4]

*Note*. Values presented are the odds ratio and corresponding 95% confidence interval. White, Black, and Other race/ethnicities are non-Hispanic. Bold font indicates that *p* < .05. Ref = reference.

#### Age

For several health effects, those who were 65 years and older were less likely to endorse that smoking can cause a given health effect as compared with those 18 to 24 years (odds ratio [*OR*] = 0.5 [0.4, 0.7] to 0.9 [0.8, 1.0]). However, those who were 65 years and older were 1.2 (1.0, 1.4) and 1.3 (1.1, 1.4) times as likely to endorse that smoking can cause bladder cancer and heart attack in nonsmokers, respectively, as compared with those aged 18 to 24 years. Those who were aged 35 to 64 years were less likely to endorse that smoking can cause lung cancer, poor circulation, mouth cancer, and harm to a fetus as compared with those aged 18 to 24 years (0.6 [0.6, 0.9] to 0.8 [0.8, 1.0]), and more likely to endorse that smoking can cause diabetes, liver cancer, stroke, heart disease, blindness, bladder cancer, and heart attack in nonsmokers (1.1 [1.0, 1.2] to 1.3 [1.1, 1.5]). Those who were aged 25 to 34 years were more likely to endorse that smoking can cause diabetes, liver cancer, stroke, blindness, lung disease in nonsmokers, and heart attack in nonsmokers (1.1 [1.0, 1.2] to 1.3 [1.1, 1.5]), as compared with those 18 to 24 years. There were no significant differences in beliefs about lung disease across age groups.

#### Sex

Compared with men, women were more likely to endorse that smoking can cause liver cancer, stroke, mouth cancer, lung disease in nonsmokers, heart attack in nonsmokers, and harm to a fetus (1.1 [1.1, 1.2] to 1.4 [1.2, 1.5]).

#### Education

For most health effects, those with at least some college education or bachelor’s/advanced degree were more likely to endorse that smoking can cause a given health effect as compared with individuals who are a high school graduate or less (1.3 [1.2, 1.5] to 4.9 [3.4, 7.2]).

#### Race/Ethnicity

Relationships between race/ethnicity and beliefs about smoking’s health effects varied. Compared with individuals who are non-Hispanic White, individuals who are non-Hispanic Black were more likely to endorse that smoking causes diabetes, liver cancer, blindness, bladder cancer, heart attack in nonsmokers, and harm to a fetus (1.1 [1.0, 1.3] to 1.5 [1.3, 1.8]), and less likely to endorse that smoking causes stroke, lung cancer, heart disease, poor circulation, mouth cancer, and lung disease (0.6 [0.5, 0.8] to 0.8 [0.7, 0.9]). Relationships were similar for Hispanics. Individuals who are Hispanic, however, were more likely than individuals who are non-Hispanic White to report that smoking causes mouth cancer (1.2 [1.1, 1.5]) and lung disease in nonsmokers due to secondhand smoke (1.5 [1.3, 1.7]). Individuals categorized as non-Hispanic Other were less likely than non-Hispanic Whites to endorse that smoking causes lung cancer, heart disease, mouth cancer, and lung disease (0.6 [0.4, 0.8] to 0.8 [0.6, 1.0]).

### Campaigns

For several health effects, frequency of viewing an antitobacco campaign was associated with beliefs about the health effects of smoking. Compared with individuals who reported never viewing a campaign, those who reported viewing a campaign often were more likely to endorse that smoking was a cause of stroke, lung cancer, heart disease, poor circulation, mouth cancer, bladder cancer, lung disease, and heart attack in nonsmokers (1.1 [1.0, 1.2] to 1.9 [1.4, 2.7]). Relationships were similar when comparing those who reported viewing antitobacco campaigns sometimes and rarely to those who reported never viewing a campaign.

### Health and Nicotine Dependence Among Smokers

#### Damage to Health

Current smokers who reported greater damage to their health as a result of tobacco use were typically more likely to endorse that smoking was a cause of a given health effect as compared with those who reported no health damage (Table 4). For all 13 health effects, compared with smokers who reported having no tobacco-related damage to their health, those who reported having a lot of damage were 1.7 [1.5, 1.9] to 7.1 [5.2, 9.8] times more likely to endorse that smoking was a cause of a given health effect. Relationships were similar when comparing smokers who reported tobacco use caused somewhat or a little damage to their health as compared with smokers who reported no damage.

**Table 4.** Correlates of Beliefs About the Health Effects of Smoking Among Current Smokers.

Characteristic	Diabetes	Liver cancer	Stroke	Lung cancer	Heart disease	Blindness	Poor circulation	Mouth cancer	Bladder cancer	Lung disease	Lung disease in nonsmokers	Heart attack in nonsmokers	Harm to fetus
Nicotine dependence													
High	**0.8 [0.8, 0.9]**	**0.9 [0.8, 1.0]**	**0.8 [0.7, 0.9]**	**0.7 [0.6, 0.9]**	**0.7 [0.6, 0.8]**	0.9 [0.8, 1.0]	**0.7 [0.6, 0.8]**	**0.7 [0.7, 0.8]**	0.9 [0.8, 1.0]	**0.8 [0.7, 1.0]**	**0.9 [0.8, 1.0]**	**0.9 [0.8, 1.0]**	**0.7 [0.6, 0.8]**
Low	Ref	Ref	Ref	Ref	Ref	Ref	Ref	Ref	Ref	Ref	Ref	Ref	Ref
Damaged health													
Not at all	Ref	Ref	Ref	Ref	Ref	Ref	Ref	Ref	Ref	Ref	Ref	Ref	Ref
A little	1.1 [1.0, 1.3]	**1.3 [1.1, 1.5]**	**1.8 [1.6, 2.1]**	**0.8 [0.7, 0.9]**	**2.0 [1.7, 2.4]**	1.1 [1.0, 1.3]	**1.8 [1.6, 3.1]**	**1.6 [1.4, 1.9]**	**0.8 [0.7, 0.9]**	**2.3 [1.8, 3.0]**	**1.3 [1.1, 1.5]**	**1.2 [1.0, 1.3]**	**1.4 [1.3, 1.7]**
Somewhat	**1.5 [1.3, 1.7]**	**1.6 [1.4, 1.8]**	**2.8 [2.4, 3.4]**	**1.4 [1.2, 1.7]**	**3.1 [2.5, 3.9]**	**1.5 [1.3, 1.7]**	**2.5 [2.1, 2.9]**	**2.4 [2.0, 2.9]**	1.1 [1.0, 1.1]	**3.6 [2.8, 4.5]**	**1.9 [1.6, 2.2]**	**1.6 [1.4, 1.9]**	**1.8 [1.5, 2.2]**
A lot	**2.2 [1.8, 2.6]**	**2.6 [2.2, 3.1]**	**6.4 [4.8, 8.6]**	**2.6 [2.0, 3.4]**	**7.1 [5.2, 9.8]**	**2.4 [2.0, 2.8]**	**5.2 [3.9, 7.0]**	**6.0 [4.4, 8.0]**	**1.7 [1.5, 1.9]**	**5.5 [3.6, 8.5]**	**3.2 [2.6, 4.1]**	**2.9 [2.3, 3.5]**	**3.4 [2.5, 4.4]**

*Note*. Regression models presented control for education, age, sex, and race/ethnicity. Values presented are the odds ratio and corresponding 95% confidence interval. Bold font indicates that *p* < .05. Sample size is *N* = 9,406.

#### Nicotine Dependence

For most health effects, smokers who reported higher levels of nicotine dependence were less likely to endorse that smoking was a cause of a given health effect as compared with smokers who reported lower levels of dependence (0.7 [0.6, 0.8] to 0.9 [0.8, 1.0]).

## Discussion

This is the first study to examine knowledge about several health effects of smoking in a nationally representative U.S. sample in over a decade. The majority (>70%) of U.S. adults correctly report that smoking can cause stroke, cancers, cardiovascular disease, and lung disease in smokers; lung disease and cardiovascular disease in nonsmokers; and harm to a fetus. Knowledge about the health effects of smoking are lowest for diabetes and blindness, however, approximately half of U.S. adults correctly report that smoking can cause these health effects. Findings from this study are consistent with prior research showing that knowledge about diseases caused by smoking varies across conditions and is typically highest for lung disease and lung cancer (Brownson et al., 1992; Oncken et al., 2005).

Despite an expanded number of communication modes and channels over time, study findings indicate enduring patterns of disparities in knowledge. Consistent with prior research, knowledge about the health effects of smoking was generally lower among respondents who were current or former smokers, older, and less educated as compared with those who were never smokers, younger, and more educated, respectively (Brownson et al., 1992; Krosnick et al., 2006; Rutten et al., 2008). For about half of the health effects examined, there were no differences between women and men in the likelihood to endorse a given health effect as caused by smoking. However, for six health effects, women were more likely to endorse a given health effect as caused by smoking as compared to men. Across race/ethnicity, knowledge about the health effects of smoking varied. Compared with non-Hispanic Whites, for most health effects there were significant differences in knowledge among respondents who were non-Hispanic Black and Hispanic. The direction of the relationship varied, however, across the health effects examined. For some health effects respondents who were non-Hispanic Black or Hispanic were more knowledgeable than non-Hispanic Whites and for other health effects they were less knowledgeable. Among respondents who were categorized as non-Hispanic Other, compared with non-Hispanic White respondents, there were significant differences in knowledge for few of the health effects examined (lung cancer, heart disease, mouth cancer, lung disease). For these health effects, respondents who were non-Hispanic Other were less knowledgeable about the health effects of smoking as compared with non-Hispanic Whites.

Smokers may be less likely to endorse that a given health effect was caused by smoking as compared with never smokers to downplay the health consequences of smoking. Bandura (1986) used the term *disengagement* to describe the denial or distortion of threatening information, such as the health effects of smoking among smokers. Current and former smokers may disengage with this information to avoid thinking about the consequences of smoking. Among current smokers, those with higher levels of nicotine dependence were also less likely to endorse that a given health effect was caused by smoking. Smokers with higher levels of nicotine dependence typically use more cigarettes per day and are less successful at quitting than those with lower levels of dependence (Baker et al., 2007). Smokers who are more addicted to nicotine may also be more likely to disengage with information about smoking’s health effects to reduce cognitive dissonance.

For all health effects examined, current smokers who reported a lot of damage to their health due to their tobacco use were more likely to endorse that a given health effect was caused by smoking as compared with current smokers who reported no health damage. Smokers who have experienced the health consequences of smoking may be more knowledgeable about smoking’s health effects because of the impact smoking has had on their lives. The present study did not examine the specific health effects of smoking respondents have experienced. Future studies should examine how knowledge about particular health effects relates to the health effects a smoker has experienced. Smokers who have experienced damage to their health due to their tobacco use may be more knowledgeable about several health effects caused by smoking, even those they have not experienced.

Findings from the present study suggest that antitobacco campaigns have been successful at increasing knowledge about the health effects of smoking. For several health effects, individuals who reported more exposure to antitobacco campaigns were more likely to endorse established health effects of smoking. Although the present study did not ask respondents about specific campaigns, the national *Tips* campaign aired during data collection for the study. *Tips* focused on several health effects of smoking, including different types of cancer, heart disease, preterm birth, and other health conditions examined in the present study (Centers for Disease Control and Prevention, 2017). Campaigns should target segments of the population with less knowledge. The CDC states that eliminating demographic disparities in tobacco use is a priority (Centers for Disease Control and Prevention, 2015). Reducing knowledge gaps across groups with different levels of formal education may reduce socioeconomic disparities in tobacco use.

There are limitations to this study. The PATH Study did not ask respondents about all possible health effects of smoking. Beliefs about the health effects of smoking may vary for other conditions. Also, although each of the health effects examined are causally related to smoking, the strength of the relationship between each health effect and smoking varies, which may impact beliefs (Andreotti et al., 2017). In addition, the wording and format used to assess health beliefs may affect participant’s responses. Although participants may believe that smoking contributes to the development of different health conditions, a participant may not agree that smoking causes the condition. In addition, the question format used to examine health beliefs in this study may result in acquiescence response bias, or the tendency to agree or disagree with all items. Studies should consider using other approaches, such as open-ended questions. In addition, the present study focused on participants’ cigarette smoker status, but did not consider use of other tobacco products (e.g., cigarillos). Beliefs about the health effects of smoking may be associated with the type of tobacco products used (O’Connor et al., 2007).

## Conclusion

Knowledge about smoking’s health effects varies across health conditions. In addition, knowledge is associated with demographic characteristics and exposure to antitobacco campaigns, as well as smokers’ health and nicotine dependence. Public health would benefit from campaigns targeting segments of the population with less knowledge about the health effects of smoking.
